# Role of Ursodeoxycholic Acid in Treating and Preventing Idiosyncratic Drug-Induced Liver Injury. A Systematic Review

**DOI:** 10.3389/fphar.2021.744488

**Published:** 2021-10-27

**Authors:** Mercedes Robles-Díaz, Lana Nezic, Vesna Vujic-Aleksic, Einar S. Björnsson

**Affiliations:** ^1^ Unidad de Gestión Clínica de Aparato Digestivo, Instituto de Investigación Biomédica de Málaga-IBIMA, Hospital Universitario Virgen de la Victoria, Facultad de Medicina, Universidad de Málaga, Centro de Investigación Biomédica en Red de Enfermedades Hepáticas y Digestivas (CIBERehd), Málaga, Spain; ^2^ Department of Pharmacology, Toxicology and Clinical Pharmacology, Faculty of Medicine, University of Banja Luka, Banja Luka, Bosnia and Herzegovina; ^3^ The Republic of Srpska Agency for Certification, Accreditation and Quality Improvement in Health Care, Banja Luka, Bosnia and Herzegovina; ^4^ Department of Internal Medicine, Landspitali University Hospital, Reykjavik, Iceland; ^5^ Faculty of Medicine, University of Iceland, Reykjavik, Iceland

**Keywords:** ursodeoxycholic acid, drug-induced liver injury, hepatotoxicity, treatment, prevention

## Abstract

**Introduction:** Treatment is generally not available for drug-induced liver injury (DILI) patients except in some specific circumstances. The management of DILI is based on the withdrawal of the responsible drug and monitoring the patients and only a few patients need to be referred to a transplant center. Some studies on the role of ursodeoxycholic acid (UDCA) in DILI have been published. The aim of this study was to perform a systematic review of the role of UDCA in the treatment and prevention of DILI.

**Methods:** A search was undertaken in PubMed, with the key words ursodeoxycholic acid, drug-induced liver injury and hepatotoxicity following the PRISMA guidelines.

**Results:** A total of 33 publications were identified: 25 case reports and 8 case series. In 18 of the 25 cases reports (22 patients), authors reported improvement of liver injury associated with UDCA therapy whereas 7 case reports did not show clinical or biochemical improvement after UDCA treatment. There were 4 studies evaluating the role of UDCA in the treatment of DILI, three prospective (one being a clinical trial) and one retrospective studies. Three studies observed liver profile improvements associated with UDCA. In addition, four studies evaluated UDCA in the prevention of DILI: one pilot study, two randomized clinical trials (RCT) and one retrospective study. Three of these studies observed a lower percentage of patients with an increase in transaminases in the groups that used UDCA for DILI prevention.

**Conclusion:** According to available data UDCA seems to have some benefits in the treatment and prevention of DILI. However, the design of the published studies does not allow a firm conclusion to be drawn on the efficacy of UDCA in DILI. A well designed RCT to evaluate the role of UDCA in DILI is needed.

## Introduction

The management of drug-induced liver injury (DILI) and liver injury due to herbal and dietary supplements (HDS) has not changed much in the last decades (Stine and Lewis, 2016). One of the utmost steps in the DILI treatment is discontinuation of the implicated drug. Apart from that, there are only a few specific drugs that have demonstrated some efficacy in the treatment of DILI. Examples of this are N-acetylcysteine in paracetamol (acetaminophen) overdose, cholestyramine in DILI caused by leflunomide and terbinafine as well as l-carnitine in valproate hepatotoxicity. Furthermore, corticosteroids are frequently used in drug-induced hypersensitivity reactions and drug-induced autoimmune hepatitis (Chalasani et al., 2014; Stine and Lewis, 2016; Andrade et al., 2019; Chalasani et al., 2021) Although acute DILI is a relatively rare condition, clinical course can be dramatic leading to a fatal outcome. Indeed, it constitutes about the 50% of all cases of acute liver failure in USA (Reuben et al., 2016).

Ursodeoxycholic acid (UDCA) is recommended by liver societies for the treatment of patients with primary biliary cholangitis (PBC) (Hirschfield et al., 2017; Hirschfield et al., 2018; Lindor et al., 2019). In a systematic review of sixteen randomized clinical trials (RCTs) including 1447 PBC patients, UDCA showed a beneficial effect on liver biochemistry levels and histological progression in comparison with placebo (Rudic et al., 2012). In PBC, the recommended dose is 13–15 mg/kg per day, orally (Hirschfield et al., 2017). Higher doses have not shown higher benefits and are associated to higher rate of side effects. At recommended doses, it is very well tolerated and side effects have very low frequency. Among described side effects are diarrhoea, slight weight gain in the first months of treatment and hair thinning (Hirschfield et al., 2017).

Although UDCA has been suggested to be of benefit in the treatment of DILI, UDCA is not recommended as a treatment of DILI according to International guidelines (Chalasani et al., 2014; Andrade et al., 2019; Chalasani et al., 2021). Literature on the effects of UDCA in DILI consists mostly of case reports and small case series and no randomized controlled trial in patients with *idiosyncratic* DILI have been performed. Although use of UDCA in patients with DILI has been reported for more than 2 decades, no formal systematic review has been undertaken to date.

The aim of the current study was to systematically review available publications exploring the role of UDCA in the treatment and prevention of DILI in clinical settings.

## Materials and Methods

A systematic review of the literature was performed with a MEDLINE search (*via* PubMed; 1946 to May 2021). This systematic review was performed following the PRISMA guidelines. The search strategy included the following main key words: “drug-induced liver injury” or “hepatotoxicity” combined with “ursodeoxycholic acid.” The review comprised any type of clinical study design with UDCA administration in DILI, therapeutic outcomes (positive or negative) and safety of UDCA. Additionally, any other relevant and specific issue related to UDCA administration in DILI were considered.

Inclusion criteria for relevant articles exploring UDCA in DILI treatment or prevention were: original studies and case reports of UDCA administration in adult or children diagnosed with liver injury induced by drugs, herbal or dietary supplements; in addition, UDCA administration for preventing DILI in patients before the onset of treatment with well-known hepatotoxic drugs. Exclusion criteria were reviews and meta-analysis, animal models, pre-clinical experimental studies with UDCA in DILI and acute liver injury due to other etiologies. References cited by the included studies and reviews were reviewed to retrieve additional references.

Each article extracted by the database search was independently screened for eligibility by different authors (R-DM, NL, V-AV) based on the criteria described above. Discrepancies were resolved by majority opinion. The work was supervised by a senior investigator (BE).

The following data were extracted from the included publications: surname of the first author, year of publication, number of patients, drug or herb responsible for DILI, DILI severity, UDCA treatment regimen, outcomes and DILI criteria when provided.

## Results

A total of 154 publications were retrieved from the database search. Of them, 52 studies were excluded based on duplications and language (case reports in other languages different from English were excluded). After screening the title and abstract, 81 records did not meet the inclusion criteria, were irrelevant records to the current study or non-original articles and were excluded. After reviewing the references of the included studies and reviews identified in the literature search, 12 additional publications were retrieved.

**FIGURE 1:**
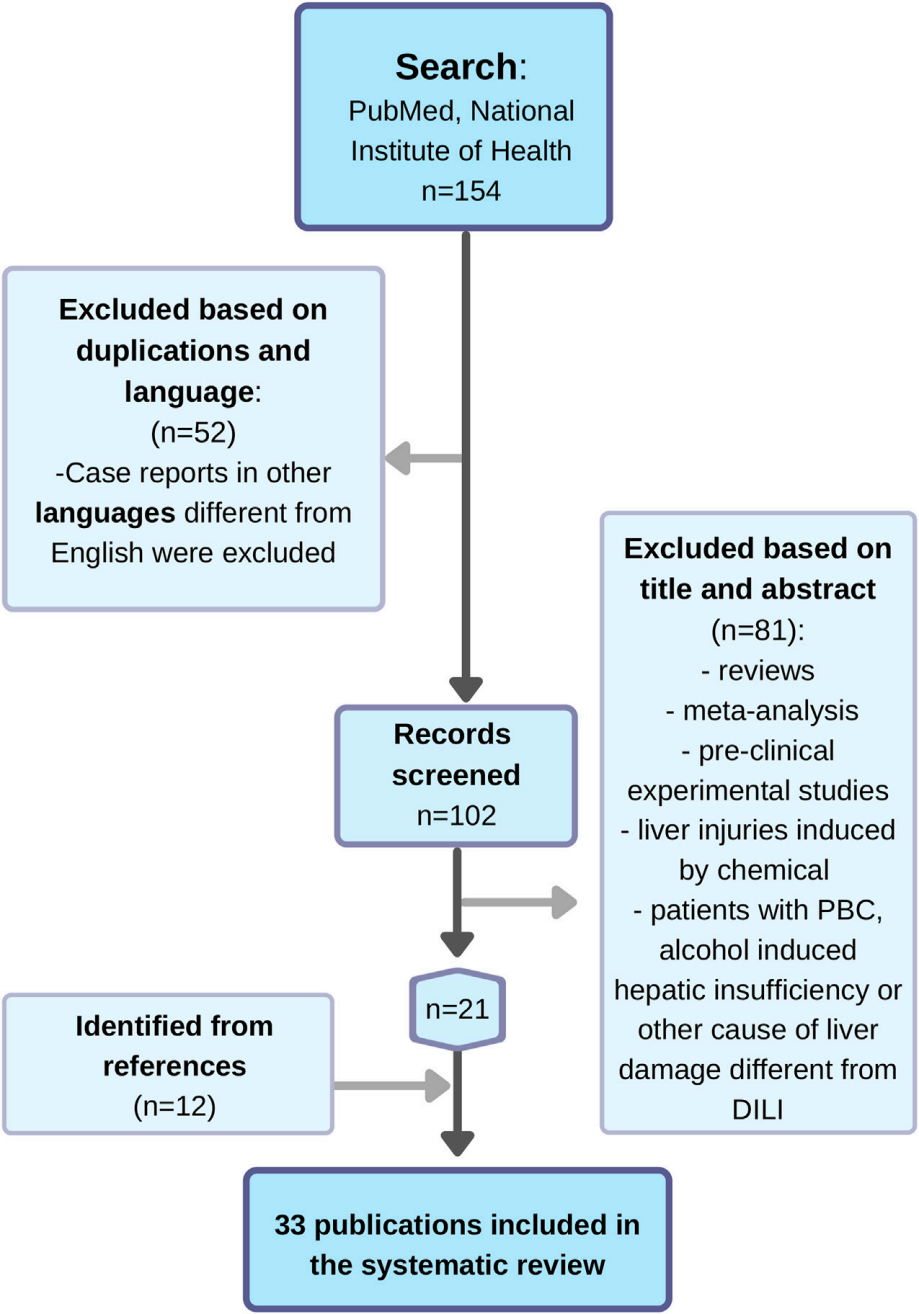
Flow chart of the search of publications about the role of UDCA in DILI treatment and prevention.

Finally, a total of 32 publications in English and one in French in which the role of UDCA for treatment or prevention of DILI was reported, were included in the current study. Eight clinical studies with observational, prospective and retrospective design as well as RCT were identified. Twenty-five case reports in English with thirty DILI patients treated with UDCA were also selected (Figure 1).

### Case Reports Dealing With Ursodeoxycholic Acid in the Drug-Induced Liver Injury Treatment or Prevention

The first case report on the role of UDCA in DILI was published in 1995 in two patients with flucloxacillin induced liver injury (Piotrowicz et al., 1995). Overall, five cases due to amoxicillin-clavulanate induced liver injury receiving UDCA (Chawla et al., 2000; Katsinelos et al., 2000; Studniarz et al., 2012; Ahmed et al., 2020) have been reported (Table 1). Furthermore, three associated to androgenic anabolic steroid (AAS) (Sánchez-Osorio et al., 2008; Krishnan et al., 2009; Stępień et al., 2015), three cases due to the combination of AAS and dietary supplements (Singh et al., 1996; El Khoury et al., 2017; Abeles et al., 2020), three due to *Centella asiatica* (Jorge and Jorge, 2005), two associated with immune check point inhibitors (Kurokawa et al., 2019; Onishi et al., 2020), one reaction each due to terbinafine (Agca et al., 2004), flutamide (Cicognani et al., 1996), ibandronate (Goossens et al., 2013), brentuximab vedotin (Neeman et al., 2019), methimazole (Gallelli et al., 2009), and Kratom that were treated with UDCA (Fernandes et al., 2019).

**TABLE 1:** Description of case reports in which ursodeoxycholic acid (UDCA) was used for DILI treatment or prevention.

Authors	Age (years)/gender (M/F)	Culprit drug in DILI	Type of liver damage	TBL and ULN (when provided)	UDCA dose/duration (when provided)	Administered drug for treatment/prevention	Clinical or biochemical improvement after UDCA administration
**1. DILI treatment**							
**a) Cholestatic and mixed cases**							
Piotrowicz et al. (1995)	a) 57/M	Flucloxacillin (2 cases)	a) Cholestatic	a) TBL peak 362 μmol/L (ULN 22 μmol/L)	a)750 mg per day/16 days	UDCA	a) Yes: 7 days after starting UDCA TBL had fallen to 198 μmol/L
	b)78/M		b) Cholestatic	b) TBL peak 252 μmol/L (ULN 22 μmol/L)	b) 750 mg per day/21 days		b) Yes: 11 days after starting UDCA TBL had fallen to 137 μmol/L
Katsinelos et al. (2000)	a) 71/M	Amoxicillin clavulanate (2 cases)	a) Cholestatic	a)TBL peak 22 mg/dl	a) 750 mg per day/1 month	UDCA	a) Yes: 10 days after starting UDCA, TBL had dropped to 12 mg/dl
	b) 81/M		b) Cholestatic	b) TBL peak 18.6 mg/dl	b)750 mg per day/2 months		b) Yes: 10 days after starting UDCA, TBL had dropped to 13.6 mg/dl
Studniarz et al. (2012)	8/M	Amoxicillin clavulanate	Cholestatic	TBL peak 11.4 mg%*	20 mg/kg per day	UDCA, steroids	Yes: improvement of cholestatic markers. Full resolution at 12 weeks of DILI onset
Chawla et al. (2000)	2/M	Amoxicillin clavulanate	Cholestatic	TBL peak 11 mg/dl	30 mg/kg per day	UDCA	No: no clinical or biochemical improvement after 4 months. Liver transplantation was successfully performed
Ahmed et al. (2020)	20/F	Amoxicillin clavulanate	Cholestatic hepatitis	TBL 6.9 mg/dl	900 mg per day/4 months	Prednisone, UDCA	Yes: following starting UDCA, clinical and laboratory improvement was observed
Krishnan et al. (2009)	38/M	17a-methyl-etioallocholan-2-ene-17b-01	Cholestatic	TBL peak 58.3 mg/dl	No data	UDCA	No: the patient remained deeply jaundiced 3 weeks after discharge. He improved after prednisone treatment
Stępień et al. (2015)	19/M	Stanozolol	Cholestatic hepatitis	TBL peak 56.54 mg/dl (ULN 1.3 mg/dl)	No data	l-ornithine l-aspartate, timonac-ic and UDCA	No: despite UDCA, TBL and INR increased. He improved after hydrocortisone treatment
Sánchez-Osorio et al. (2008)	29/M	Mesterolone, testosterone undecanoate, nandrolone undecanoate, oxymetholon, stanozolol and testosterone	Cholestatic	TBL 29 mg/dl	15 mg/kg per day/4 months	UDCA	Yes: just after the beginning of UDCA treatment, TBL started to decrease and dropped near 50% in 1 month
Singh et al. (1996)	24/M	Nandrolone, stanozolol, ornithine, cola nut extract, Ma Huang, carnitine, chromiun picolinate, mixure of aminoacids and protein supplements	Mixed	TBL peak 53 mg/dl (ULN 1 mg/dl)	900 mg per day/at least 6 months since onset of jaundice	UDCA	Yes: TBL decreased 59% in 1 month and an additional 64% 2 weeks later
Kurokawa et al. (2019)	48/M	Pembrolizumab	Cholestatic	TBL peak 5.4 mg/dl	11 mg/kg per day	UDCA, steroids	Yes: ALP and GGT dropped 70% in 2 weeks
Neeman et al. (2019)	67/M	Brentuximab vedotin	Cholestatic	TBL peak 600 μmol/L	15 mg/kg per day	UDCA	No: TBL progressively increased despite UDCA treatment. Patient death due to a sepsis of unknown origin
Gallelli et al. (2009)	54/M	Methimazole	Cholestatic	TBL 4.4 mg/dl (ULN 1 mg/dl)	No data	UDCA, cholestyramine and chlorpheniramin	Yes: resolution of clinical and biochemical alterations 5 days after
Fernandes et al. (2019)	52/M	Kratom	Cholestatic	TBL peak 28.9 mg/dl	1800 mg per day/about 2 months	UDCA	Yes: rapid decreased in TBL in 2 weeks (from 28.9 mg/dl to 4 mg/dl)
**VBDS**							
Smith et al. (2005)	10/M	Amoxicillin clavulanate	Cholestatic	TBL peak 26.5 mg/dl (ULN 1.2 mg/dl)	15 mg/kg/day (increased to 30 mg/kg/day after 1 month, and to 45 mg/kg after 6 weeks)	UDCA	Yes: 2 weeks after UDCA was increased to 45 mg/kg/day a significant improvement in TBL was observed
O’Brien et al. (1996)	58/F	Haloperidol, prochlorperazine, amitriptyline and phenytoin	Cholestatic	TBL 11.8 mg/dl (ULN 1 mg/dl)	600 mg/day during 2 years, 300 mg/day 6 more months	UDCA	Yes: 50% reduction in ALP, GGT, ALT and AST 6 weeks after the beginning of UDCA
Tejedor-Tejada et al. (2019)	39/F	Sertraline	Cholestatic	Exact data not provided	1800 mg per day/3 months and 600 mg per day/about 3 more months	UDCA, steroids	No: cholestasis persisted despite UDCA during 3 months. The patient was successfully treated with plasmapheresis
**b) Hepatocellular cases**							
El Khoury et al. (2017)	35/M	Stanozolol, trenbolone, testosterone propionate, T3 liothyronine, creatine, conjugatedlinoleic acid with olive oil, and amino acids	Hepatocellular	TBL peak 38.6 mg/dl (ULN 1 mg/dl)	15 mg/kg/day	sodium bicarbonate, albumin, UDCA and hydroxyzine	No: the patient status got worse (increase in bilirubin) Patient improved after several sessions of plasma exchange
Abeles et al. (2020)	36/M	Patient 2: Creatine, “Winter Cherry” (Ashwagandha) and methyldrostanolone	Hepatocellular	TBL peak 768 μmol/L	No data	Chlorphenamine, UDCA, hyroxyzine and NAC	No: increase in bilirubin
							Patient improved (decrease in bilirubin) just after starting prednisolone
Jorge and Jorge (2005)	a)61/F	*Centella asiatica* (3 cases)	a) Hepatocellular	a) TBL 4.23 mg/dl	a) and b)10 mg/kg per day/2 months	UDCA	Yes: a) and b) 2 months and c) 1 month after starting UDCA the patients were asymptomatic and anicteric
	a) 52/F		b) Hepatocellular	b) TBL 19.89 mg/dl	c) 10 mg/kg per day/1 month		
	a) 49/F		c) Hepatocellular	c) TBL 3.9 mg/dl			
Onishi et al. (2020)	68/M	Nivolumab	Hepatocellular	TBL 0.3 mg/dl	10 mg/kg per day/3 months	UDCA	Yes: rapid decrease in ALP from 960 to 240 U/L in about 40 days just after UDCA initiation
Agca et al. (2004)	56/F	Terbinafine	Hepatocellular	TBL peak 60.2 mg/dl	15 mg/kg per day/15 weeks	UDCA	Yes: 2 weeks after UDCA initiation, pruritus was diminished and liver function test values started to return to normal
Cicognani et al. (1996)	83/M	Flutamide	Hepatocellular	TBL peak 24.5 mg/dl (ULN 0.8 mg/dl)	12 mg/kg per day/not provided	UDCA	Yes: during the following month after UDCA initiation the clinical condition and biochemical results progressively improved
Goossens et al. (2013)	61/F	Ibandronate	Hepatocellular	TBL 137.4 μmol/L (ULN 19 μmol/L)	10 mg/kg per day	UDCA	Yes: liver profile normalized after UDCA initiation
**VBDS**							
Li et al. (2019)	6/M	Amoxicillin and naproxen	Hepatocellular	TBL peak 349 μmol/L (ULN 21 μmol/L)	15 mg/kg/day (increased to 40 mg/kg/day after 6 weeks)	UDCA, S-adenosyl-l-methionine, alprostadil, Pien TzeHuang (traditional Chinese medicine), and a low fat diet	Yes: initial increase in liver test but further decrease after UDCA dose increase
**2. DILI prevention**							
Ito et al. (2014)	a) 64/F	a) A bosentan DILI case had the drug stopped and readministered in lower doses together with UDCA	a) Cholestatic	NA	300 mg/day (increased to 600 mg/day after 2 years in first case and after 3 months in second case)	UDCA	Yes: no new increases in liver test were observed after re-administration of bosentan even after increasing to normal dose (dose that previously had cause DILI) when it was administered simultaneously with UDCA
	b) 69/F	b) In a bosentan DILI case the dose was reduced and administered together with UDCA	b) Cholestatic				

Abbreviations: ALP: alkalin phosphatase; ALT: alanine aminotransferase; AST: aspartate aminotransferase; ERCP: endoscopic retrograde cholangiopancreatography; F: female; GGT: gamma glutamyl transferase; INR: international normalized ratio; M: male; NAC: N-acetylcysteine; TBL: total bilirubin level; UDCA: ursodeoxycholic acid; ULN: upper limit of normal; VBDS: vanishing bile duct syndrome.

Type of liver damage was based on ratio (R) when provided. R = ALT/ULN divided by ALP/ULN being R ≥ 5 hepatocellular, R ≤ 2 cholestatic and R > 2 and <5 mixed. When R was not provided or there were no data to calculate it, type of liver damage was based on liver biopsy findings or author description.

a TBL expressed as mg% in the source manuscript.

DILI cases have been adjudicated by the original authors.

Clinical improvement after UDCA treatment was observed in almost all amoxicillin-clavulanate cases (4 out of 5) but only 2 out of 6 cases due to AAS with or without dietary supplements. Among the other nine DILI reports, therapeutic response to UDCA was found to be positive in all except for one associated with brentuximab vedotin (Neeman et al., 2019) (Table 1).

In addition, there was one case of vanishing bile duct syndrome due to amoxicillin-clavulanate (Smith et al., 2005) and another one due to a combination of haloperidol, prochlorperazine, amitriptyline and phenytoin (O’Brien et al., 1996). In both of these reports important improvement was observed after starting treatment with UDCA. Another case of vanishing bile duct syndrome after the use of amoxicillin and naproxen in a child has been reported. In this case a slow improvement was seen only after increasing UDCA (40 mg/kg per day, orally) and adding, S-adenosyl-l-methionine, alprostadil, Pien TzeHuang (traditional Chinese medicine), and change in the fat diet to the treatment (Li et al., 2019). An additional case of sertraline-induced vanishing bile duct syndrome did not show improvement of liver tests after treatment with steroids and UDCA (Tejedor-Tejada et al., 2019) (Table 1).

Finally, there were two case reports of bosentan inducing DILI (Ito et al., 2014). When the episodes of DILI were resolved, bosentan, in lower doses, was restarted in the two cases together with UDCA. Bosentan treatment could then be continued without new DILI episodes. If it was adaptation or the preventive effect of UDCA is impossible to make any conclusions on. The total number of retrieved cases reports are presented in the Table 1, showing the author, patients’ age and gender, implicated drug, total bilirubin (TBL) peak level, UDCA dose and treatment duration, UDCA (and other drugs if any) administration for treatment or prevention purpose in DILI, type of liver damage and suggested benefit/therapeutic outcome.

No differences in response to UDCA were found regarding the cases had hepatocellular or cholestatic type of liver injury: 13/18 patients among cholestatic DILI cases and 8/10 among hepatocellular ones showed improvement after UDCA administration (Table 1).

Regarding the severity of DILI and implicated drug, 4 out of 7 cases who did not respond to UDCA were DILI cases due to AAS (2 hepatocellular and 2 cholestatic cases) with high TBL level (ranging from 38.6 to 58.3 mg/dl).

DILI diagnosis was performed by original authors. Most of DILI case reports had an extensive panel of serological, autoantibodies and image test and even biopsy findings to rule out alternative causes of liver injury before the DILI diagnosis, however only in two cases Roussel Uclaf Causality Assessment Method (RUCAM) (Danan and Benichou, 1993) was applied for DILI assessment (Gallelli et al., 2009; Abeles et al., 2020) (4 points, possible and 6 points, probable association to the drug, respectively) and in one case Naranjo scale (Naranjo et al., 1981) was applied (Agca et al., 2004) (probable relationship to the drug). Even assuming all of them are true DILI cases, none of the authors compared the time to resolution of their cases with other DILI cases not treated with UDCA. Despite that, most authors considered that the treatment with UDCA benefited the patients and that UDCA helped to improve and or resolve the liver damage in these patients.

### Clinical Studies With Ursodeoxycholic Acid in the Drug-Induced Liver Injury Treatment or Prevention

#### Therapeutic Efficacy of Ursodeoxycholic Acid in Drug-Induced Liver Injury

Eight retrieved clinical studies with UDCA administration in DILI are presented in Table 2, with the following characteristics: author, type of the study, number of patients with DILI diagnosis and UDCA dosage, suspected/implicated drug for DILI, UDCA administration for treatment or prevention purpose in DILI, and suggested benefit/therapeutic outcome. DILI criteria of each included study, when provided, are also detailed in Table 2. Four studies with 91 DILI patients receiving UDCA evaluated its role in DILI treatment. Three were carried out in adult patients, while one was in pediatric population. Four other studies with 414 patients evaluated UDCA in DILI prevention, two in children and two in adult populations.

**TABLE 2:** Description of prospective and retrospective studies evaluating the role of ursodeoxycholic acid in the treatment and prevention of DILI.

Authors/year	DILI criteria	Type of study	Number of DILI patients and UDCA dosage	Culprit drug	Administered drug for treatment/prevention DILI	Benefit suggested
**Treatment**						
Wree et al. (2011)	DILI criteria: not provided.	Prospective DILI cohorts compared with historical controls from the DILIN network	15 adult DILI patients	Conventional medications (non-anabolic steroids) *n* = 10	Corticosteroid and UDCA	Yes (significantly more rapid decrease in TBL levels)
	Included patients: severe liver damage or acute liver failure induced by drugs or HDS		-9 with corticosteroids 2–5 mg/kg/day step-down therapy + UDCA	Anabolic steroids *n* = 5		
			-6 steroid pulse therapy (15–20 mg/kg/day) for 3 days + UDCA (750–1,500 mg/day)			
Lang et al. (2019)	ALT >5 ULN, ALP >2 ULN or TBL> 2ULN.	Prospective pilot study not compared with placebo	27 out of 285 adult patients with anti mycobacterial drugs, developed DILI and were treated with UDCA (250–500 mg/8 h with a later reduction to 250–500 mg/day)	Anti mycobacterial drugs	UDCA	Yes (normalization or significant reduction of ALT, AST, ALP and/or TBL)
		Compared with excluded DILI group due to CLD and others not treated with UDCA				
Saito et al. (2016)	ALT> 2ULN	Retrospective	71 DILI out of 389 adult patients with antiTBC drugs. 27, included in the analysis, were treated with hepatoprotective drugs (dosage of UDCA not provided)	AntiTBC drugs	UDCA, stronger neo-minophagen C, and glycyrrhizin	No
		Comparison between patient treated or not with hepatoprotective drugs				
Asgarshirazi et al. (2015)	ALT, AST and GGT > 2 ULN	Clinical trial	22 young DILI patients (children age range 4 months-3 years) were treated with UDCA (10–15 mg/kg/day)	Anticonvulsants	UDCA	Yes (significant decline in transaminase levels despite maintenance of anticonvulsant treatment)
		No control group				
**Prevention**						
Bordbar et al. (2018)	NA	Randomized clinical trial with control group	80 children (age range 2–18 years) on chemotherapy randomized to 4 groups of preventive treatment (UDCA dosage 15 mg/kg/day)	Chemotherapy	vit E, UDCA, Vit E + UDCA or control group	No
		Iranian Registry of Clinical Trials: IRCT2013120515666N1				
Mohammed Saif et al. (2012)	NA	Pilot, randomized open label, parallel control study	39 children (age range 2–18 years) with ALL treated with chemotherapy. 19 were randomized to receive UCDA (10–15 mg/kg/day), 20 to control group (without UDCA)	Chemotherapy	UDCA	YES ( a trend toward decreased levels of transaminases compared with control group)
Kojima et al. (2002)	NA	Retrospective study	Out of 181 adult patients on flutamide treatment, 70 were also treated with UDCA (375 mg/day) for DILI prevention	Flutamide	UDCA	Yes (lower percentage of patients with increases in transaminases in the UDCA group compared with patients without UDCA)
Salmon et al. (2001)	NA	Pilot study	14 patients out of 114 with Alzheimer disease in treatment with tacrine, received also UDCA (13 mg/kg/day) for DILI prevention	Tacrine	UDCA	Yes (higher percentage of normal ALT in the UDCA group compared with patients without UDCA)

Abbreviations: ALP: alkaline phosphatase; ALT: alanine aminotransferase; AST: aspartate aminotransferase; CLD: chronic liver disease; DILI: drug-induced liver injury; DILIN: Drug-Induced Liver Injury Network; GGT: gamma glutamil transferase; HDS: herbal and dietary supplements; TBC: tuberculosis; UDCA: Ursodeoxycholic acid; ULN: upper limit of normal.

In a study carried out by Wree et al. (2011), 15 patients with severe liver damage or acute liver failure induced by drugs or HDS were treated with corticosteroids 2–5 mg/kg/day [step-down therapy (N = 9)] or steroid pulse therapy (15–20 mg/kg/day) for 3 days/pulse (N = 6), and UDCA 750–1,500 mg/day (concomitantly in both groups) for several weeks (range 4–10 weeks). Aminotransferases and TBL levels decreased to <50% of peak values within 2 weeks, and normalized within 4–8 weeks. These patients in comparison with historical controls from the Drug-Induced Liver Injury Network (DILIN) were found to have a significantly more rapid decrease in TBL levels (Wree et al., 2011) (35).

Lang et al. (2019) evaluated the utility of UDCA in patients with tuberculosis (TBC) and DILI [defined as an increase in alanine aminotransferase (ALT) > 5 upper limit of normal (ULN), alkaline phosphatase (ALP) > 2 ULN or TBL> 2ULN] due to anti-TBC drugs in a prospective study but uncontrolled design. Thirty-nine out of 285 (9.5%) patients receiving anti-TBC drugs developed clinically relevant hepatotoxicity. Twelve were excluded from the analysis. Causes of exclusion were chronic liver disease, malnutrition, advanced chronic kidney disease, intolerability of anti-TBC drugs other than DILI and taking other potentially hepatoprotective drugs. In total 27 patients with DILI were treated with UDCA (250–500 mg/8 h with a later reduction to 250–500 mg/day until normalization of liver profile); drug dose was not reduced in any of these patients. Twenty-one patients showed a normalization in their liver profile, and 5 patients an important improvement in the liver tests, but only in one patient liver tests did not change. By comparison, all 12 patients excluded from the UDCA study showed no improvement or even progressed in the parameters of liver injury. However, this might be due to a selection of a sicker group of patients excluded from the UDCA study.

A Japanese retrospective study evaluated the role of hepatoprotective drugs [UDCA, stronger neo-minophagen C, and glycyrrhizin] in patients with DILI induced by anti-TBC drugs (Saito et al., 2016). In total 389 patients with active TBC were included in this study. Out of them, 43 patients developed mild DILI (ALT > 2 ULN and < 3 ULN), none of them stopped the anti-TBC treatment, and only 10 patients were treated with hepatoprotective drugs. The average time to normalization of aminotransferases was longer in the subgroup with hepatoprotective drugs. A moderate DILI (ALT between 3 and 5 ULN) developed in 5 patients (not included in the analysis due to the low number of patients) while 23 patients developed severe DILI (ALT > 5 ULN) and therefore discontinued the treatment. Among those with severe DILI, 17 received some hepatoprotective drugs. Results showed no differences in the average time to aminotransferases normalization in the group with severe DILI. However, the data analysis did not provide a clear data regarding the duration of treatment and dosage of UDCA. Moreover, the study failed to distinguish between the patients who were treated with UDCA or with other hepatoprotective drugs. These limitations made it impossible to conclude if a single or combined treatment with hepatoprotective drugs were the culprit of the apparent lack of therapeutic benefit of these therapies.

Asgarshirazi et al. (2015) in an uncontrolled clinical trial described pediatric patients with significant elevation (>2 ULN) in AST, ALT and gamma-glutamyltransferase (GGT) secondary to anticonvulsants confirmed at monthly follow up. Anticonvulsant drug doses were reduced or the treatment changed if possible and those patients who did not resolve liver tests despite that, were treated with UDCA (10–15 mg/kg/day) for at least 24 weeks (*n* = 22). The results showed a significant improvement of liver tests after treatment with UDCA although due to uncontrolled design of this study a firm conclusion cannot be drawn.

#### Role of Ursodeoxycholic Acid in Drug-Induced Liver Injury Prevention

The potential benefits of UDCA have been analyzed not only in the treatment of DILI but also for potential prevention. A Turkish study evaluated the effect of UDCA and vitamin E in preventing liver fibrosis in patients receiving chemotherapy (Bordbar et al., 2018). A total of 80 pediatric patients with B-cell acute lymphoblastic leukemia were randomized into 4 groups of treatment: vitamin E, UDCA (15 mg/kg/day), vitamin E + UDCA or control group (without UDCA and/or vitamin E except for chemotherapy). No differences were found regarding liver fibrosis among the groups. In the group treated with UDCA the results showed a trend to decrease in aminotransferase levels during the study and at 6 months after discontinuation of UDCA compared with baseline. Slight increase in TBL was found in the group treated with vitamin E only over the study period compared to the baseline levels.

A similar prospective randomized, open labelled parallel control pilot study was carried out in 39 pediatric patients receiving chemotherapy for acute lymphatic leukemia (Mohammed Saif et al., 2012). Considering the hepatotoxicity potential of various chemotherapy protocols, UDCA was investigated as hepatoprotective agent in terms of its safety and efficacy. Subjects were randomized into two groups according to the UDCA therapy allocation. The UDCA group consisted of patients who received UDCA (10–15 mg/kg/day) concomitantly with chemotherapy for 6 months, and were followed up for additional 3 months after UDCA discontinuation. The control group consisted of patients who received chemotherapy without UDCA and were followed up for 9 months in total (over treatment period and 3 months after). The results showed that in patients concomitantly treated with UDCA there was a trend toward decreased levels of aminotransferases. Also, there was a new increase in ALT after stopping UDCA during a follow-up period in the UDCA group.

A retrospective study evaluating the role of UDCA in 181 patients with prostate cancer treated with flutamide for preventing DILI has been undertaken (Kojima et al., 2002). Out of them only 70 patients were treated with UDCA (375 mg/day). A mild and moderate increase in hepatic aminotransferases were reported in 11.4% patients treated with UDCA but in 32.4% without it, which was a significant difference. Similarly, a severe increase in aminotransferases and clinically serious liver injury were only documented in patients without UDCA in comparison to those receiving UDCA in prevention dosage regime.

A pilot open label (Salmon et al., 2001) study of 114 patients with Alzheimer disease was also carried out. All the patients were treated with tacrine, out of whom, 14 patients were also treated with UDCA (13 mg/kg/day for 105 days). In 93% of patients in the UDCA group had normal ALT at the end of the study compared to 69% in those who did not received UDCA. Moderate hepatotoxicity (ALT between 1 and 3x ULN) only occurred in the control group (25%) while number of patients with ALT> 3xULN were similar in both groups (7 vs. 6%, respectively). Based on these results, the authors concluded that UDCA could prevent moderate tacrine-induced hepatotoxicity (Salmon et al., 2001).

## Discussion

Ursodeoxycholic acid has been used empirically for a long time in cholestatic DILI with the aim to shorten the time to resolution.

The potential benefits of UDCA in DILI could be explained by the pathophysiological mechanism of UDCA in the liver. There is growing number of mechanistic studies conducted on UDCA that have yielded convincing evidence regarding UDCA-mediated hepatoprotection in cholestatic liver disease. In addition, cholestatic mechanisms often contribute to DILI, even when the clinical presentation is hepatocellular. In fact, DILI developed by bile salt export pump inhibition may present as hepatocellular pattern of liver injury, resulting in an important hepatocyte death and high elevation of aminotransferases and minimal increase in ALP and TBL (PSTC BSEP Webinar Series, 2006). For this reason, the mechanisms underlying the benefit of UDCA in cholestatic disease may reasonably apply in hepatocellular and mixed DILI.

Due to its hydrophilic nature and lack of membrane-disrupting properties UDCA seems to be nontoxic to hepatocytes even in high concentrations (Paumgartner and Beuers, 2004; Ashby et al., 2018). The exception was though found in a study of high dose UDCA in patients with primary sclerosing cholangitis (Lindor et al., 2009). Potential therapeutic mechanism of UDCA may be related to its ability to regulate hepatocytes apoptosis and survival, to exert membrane-stabilizing, antioxidative, and immunomodulatory effects (Mohammed Saif et al., 2012).

*In vitro* studies showed that UDCA affects expression of genes directly involved in the hepatocytes cycle and apoptosis (Castro et al., 2005; Ali et al., 2020), oxidative cell damage (Lapenna et al., 2002) and suppression of hepatic lipid peroxidation in experimental cholestatic liver disease (Ljubuncic et al., 2000).

Recently, work by Li et al. (2020) demonstrated a broad therapeutic potency of UDCA in the setting of liver injury induced by known hepatotoxic arsenious acid. UDCA was found to activate Nuclear factor erythroid-2-related factor 2 (Nrf2) pathway that plays an essential role in cellular defense against apoptosis, oxidative stress or promotes cell survival by activating antioxidant cascades (Li et al., 2020). Finally, it has been described that UDCA binds to glucocorticoid receptor that could mediate its anti-inflammatory and immunomodulatory effects (Miura et al., 2001). However, the relevance of these proven UDCA effects *in vitro* needs to be confirmed *in vivo* and in the clinical setting.

Experimental results showed that antiapoptotic (Amaral et al., 2007) and antioxidative (Uraz et al., 2008) properties of UDCA might explain hepatoprotective effects of UDCA against methotrexate induced liver injury in some clinical studies (Mohammed Saif et al., 2012; Bordbar et al., 2018). Similarly, in animal models of DILI induced by ceftriaxone (Alhumaidha et al., 2014) or combination of anti-TBC drugs isoniazid plus rifampicin (Chen et al., 2011), hepatoprotective effects of UDCA were shown by preservation of liver histology, decrease in liver enzymes, elevation of antioxidant glutathione content and decrease of malondialdehyde and nitric oxide.

Despite the potential role of UDCA in prevention or treatment of DILI in humans, early and sensitive biomarkers of its therapeutic response are still lacking. In a recent metabolomic study, it has been shown that microRNA-122 (miR-122) could be considered as an early biomarker of DILI (Lee et al., 2017). The miR-122 is the most prevalent miRNA (approximately 70%) in the adult liver (Bandiera et al., 2015), and is involved in regulation of liver transcription factors and maintaining hepatocyte life cycle (Vincent and Sanyal, 2014). The results also demonstrated that UDCA significantly decreased miR-122 (Kim et al., 2019) levels indicating UDCA as an potential agent in ameliorating or preventing DILI in humans, and miR-122 as a potentially reliable early biomarker of DILI. UDCA seems to prevent apoptosis in hepatocytes at the mitochondria level and protect cholangiocytes against the toxic action of endogenous bile acids (Beuers, 2006; Beuers et al., 2015).

Although most studies analyzed in the current systematic review suggest a positive therapeutic and protective role of UDCA, it does not allow us to conclude on definite dose regime of UDCA in treating or preventing DILI. We found that the therapeutic efficacy of UDCA in the treatment of DILI was beneficial with lower dose range of 250–500 mg/day as well as with 750–1,500 mg/day (up to 45 mg/kg/day) over at least 4 weeks up to several months depending of the causative drug and severity of DILI, administered as a single treatment or concomitantly with other hepatoprotective agents. Similarly, therapeutic outcomes in the DILI prevention with UDCA were inconsistent, and most of them were achieved with wider dose range of UDCA (most commonly 10–15 mg/day) over the entire treatment with known hepatotoxic drugs, but the dosing was frequently increased based on assessed liver tests.

Our systematic review had certain limitations. These clinical studies show large heterogeneity in methodology and the criteria for DILI diagnosis, the time of initiation and duration of treatment, and UDCA dose regime in terms of single and daily doses. Furthermore, design of the studies reviewed either retrospective or prospective was mostly without a control group. Numerous anecdotal case reports and most of the case series had serious methodological weaknesses which makes it almost impossible to conclude on the efficacy of UDCA in patients with DILI. Also, making definite conclusion on the efficacy of UDCA in DILI treatment is difficult to distinguish due to often concomitant administration of potential hepatoprotective agents.

DILI is a rare adverse effect and resolves in most cases spontaneously without much morbidity and a very low mortality. However, in some patients, recovery is slow and they can suffer from itching, general weakness and low energy level for many weeks or months.

Given the state of the existing knowledge based on the extensive preclinical research where therapeutic potency of UDCA in DILI has been demonstrated, it is necessary to explore further mechanisms of UDCA in clinical studies. In order to definitely elucidate UDCA efficacy in the treatment of patients with DILI, a randomized clinical trial controlled with placebo should be initiated in multicenter settings and last long enough to reach the necessary number of patients. The groups should be evenly distributed by type of liver injury (hepatocellular, cholestatic and mixed) and by severity (mild, moderate severe) as defined by a Expert International Consensus Meeting in 2011 (Aithal et al., 2011). The main endpoints need to be significant decline (i.e., 50% vs. baseline) of liver enzymes (ALT, AST, ALP and TBL), time to complete normalization of liver enzymes and rate of survivals as well as DILI relapse rate after UDCA withdrawal and its safety profile. Due to heterogeneity of the affected patients, inclusion criteria should be focused on the patients with clinical diagnosis of DILI defined as ALT ≥ 5 x ULN, ALP ≥ 2 x ULN or ALT ≥ 3xULN plus TBL >2 xULN (Aithal et al., 2011) and RUCAM score (Danan and Benichou, 1993), at least, possible (≥3).

In this review, we summarized the history and current status of UDCA use in prevention and treatment of DILI, explored the culprit drugs and the patients who may benefit from UDCA therapy, and presented weakness of the available results of UDCA treatment of DILI. Relatively often empiric use of UDCA in DILI appears to be safe and leads to a reduction in bilirubin and transaminase levels. Therefore, our current observations promise a strong base for in-depth clinical investigations into its efficacy (both in prevention and treatment) in patients with confirmed DILI.

## Data Availability

The original contributions presented in the study are included in the article/Supplementary Material, further inquiries can be directed to the corresponding author.

## Abbreviations

AAS, androgenic anabolic steroid; ALP, alkaline phosphatase; ALT, alanine aminotransferase; AST, aspartate aminotransferase; CLD, chronic liver disease; DILI, drug-induced liver injury; DILIN, Drug-Induced Liver Injury Network; ERCP, endoscopic retrograde cholangiopancreatography; GGT, gamma-glutamyl transferase; HDS, herbal and dietary supplements; INR, international normalisation ratio; miR-122, microRNA-122; NAC, N-acetylcysteine; PBC, primary biliary cholangitis; RCT, randomized clinical trials; RUCAM, Roussel Uclaf Causality Assessment Method; TBC, tuberculosis; TBL: total bilirubin level; UDCA, ursodeoxycholic acid; ULN, upper limit of normal; VBDS: vanishing bile duct syndrome.
